# Large intra-abdominal mucinous cystic adenoma: is it of ovarian or mesenteric origin

**DOI:** 10.11604/pamj.2020.36.122.21642

**Published:** 2020-06-25

**Authors:** Radisnay Guzmán Lambert, Barbara Yordanis Hernandez Cervantes, Mariuska Rodríguez Gonzalez, Duniesky Martínez Lopez, Frank Edwin

**Affiliations:** 1 Department of Surgery, School of Medicine, University of Health and Allied Sciences, Ho, Volta Region, Ghana,; 2 Department of Internal Medicine, School of Medicine, University of Health and Allied Sciences, Ho, Volta Region, Ghana

**Keywords:** Cystadenoma, histophatology, conundrum

## Abstract

A 54-year-old female presented with a six year history of increasing abdominal swelling and discomfort and two months of intermittent constipation and difficulty with micturition. She was referred from the gynecological service having been investigated for a pelvic pathology without any positive findings. Her medical history was otherwise unremarkable. Physical examination revealed a non-tender intra-abdominal mass extending from epigastrium to the pelvis with a smooth surface. A large intra-abdominal multi-loculated cyst, separate from the ovaries, was seen on imaging. At laparotomy, the cystic tumour was discovered to arise from the mesentery of the terminal ileum and was resected en bloc. Histopathology revealed the tumour to be a benign mucinous cystadenoma, possibly of ovarian origin. This report aims to raise awareness of the difficulty of distinguishing ovarian from extra-ovarian mucinous cystadenomas on histopathological examination alone.

## Introduction

Intra-abdominal mucinous cystic neoplasms commonly arise from the ovaries. When they are large and seemingly involve both the mesentery and adnexa, determination of their tissue of origin becomes problematic. Mucinous cystadenomas arising from extra-ovarian sites are very rare. They are mucin-producing cystic tumors with an ovarian-like stroma that may arise from the pancreas, liver, mesentery and spleen. Mesenteric cysts are so rare they are not usually considered in the differential diagnosis of intra-abdominal masses. Prior to 2009, there were only thirteen reported cases of mucinous cystadenoma (MCA) involving the mesentery [1]. We report a patient who presented with a large intra-abdominal cystic mass of debatable origin: the surgical findings supported a mesenteric origin but the histopathology report suggested an ovarian mucinous cystadenoma.

## Patient and observation

**Figure 1:**
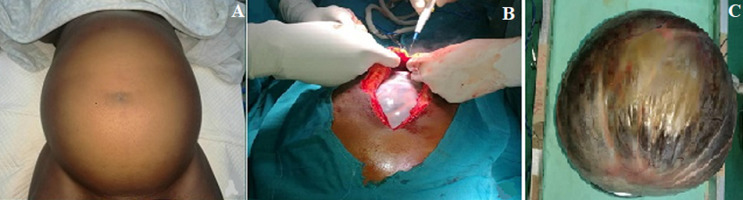
(A) abdomen prior to surgery; (B) the cyst at laparotomy; (C) the cyst after resection en-bloc

The patient is a 54 year-old lady who presented with a six year history of increasing abdominal swelling and discomfort and two months of intermittent constipation and difficulty with micturition. The swelling and discomfort began in the lower abdomen. She was referred from the gynecological service having been investigated for a pelvic pathology without any positive findings. Her medical history was otherwise unremarkable. Physical examination revealed a non-tender intra-abdominal mass extending from epigastrium to the pelvis with a smooth surface (Figure 1). The mass was mobile and dull to percussion. Rectal examination was normal. Her hemoglobin was 12.7g/dl, white cell count 3.9 x 10^9^/l, and ESR of 52mm fall/hour. Abdominal ultrasonography showed a large multi-loculated cystic intra-abdominal mass measuring 18.5cm in largest dimension. Both ovaries and kidneys were reported as normal. Computed tomography scan of the abdomen confirmed the ultrasound findings. The patient was prepared for exploratory laparotomy. Laparotomy revealed a large cystic mass (Figure 1) 20cm in widest dimension arising from the base of the mesentery of the terminal ileum and attached to the right adnexa. Both ovaries were intact. The mass was dissected and resected en-bloc (Figure 1) with the right adnexa for histological examination. The specimen weighed 6kg and measured 20cm x 19cm x 7cm. Histological examination revealed a multi-loculated cyst containing gelatinous material. The wall consisted of tall non-ciliated columnar cells with basal nuclei and abundant intracellular mucin consistent with mucinous cystadenoma. The histopathology report suggested this was an ovarian mucinous cystadenoma.

## Discussion

Cysts arising from the mesentery and retroperitoneum are extremely rare lesions identified in 1 of 105,000 admissions in adults [2]. The cysts are most common in the small-bowel mesentery, especially the ileum. Their presentation varies but as in the case presented here, unspecific and chronic symptoms involving abdominal pain, distention, a palpable mass which is movable transversely but not longitudinally, gastrointestinal and urinary obstruction are common [2-4]. Preoperative imaging usually involves ultrasonography and computed tomographic studies of the abdomen. Ultrasonography is the preferred diagnostic method [5] Other imaging modalities such as plain abdominal radiographs, computed tomography (CT) studies and magnetic resonance imaging (MRI) may be useful in selected cases. When bowel involvement is uncertain, more advanced imaging techniques involving barium enema, CT and MRI could be more informative [6].

Of the thirteen cases of MCA of the mesentery reported prior to 2009, preoperative imaging was inconclusive in nine cases, suggested ovarian origin in four cases and mesenteric origin in only one case [1]. Because ovarian MCAs are relatively more common, extra-ovarian MCAs are commonly mistaken for the ovarian variety. Even more interesting is the fact that ovarian and extra-ovarian MCAs are essentially identical in histological features [7]. Histological examination alone is thus unsatisfactory in determining the origin of the tumor. The similarities between ovarian and extra-ovarian MCAs suggest a common pathway of development [8]. The cyst wall of extra-ovarian MCAs is lined by mucin-secreting flat, cuboidal or columnar epithelium associated with an underlying sub-epithelial ovarian like stroma. This is the same as may be found in ovarian MCA with the important distinction that when normal ovarian tissue is present, its stroma is distinctly different from the stroma of the septa found within the cyst [7]. The origin of extra-ovarian MCAs has been attributed to implanted or ectopic ovarian tissue, supernumerary ovaries, or mono-phyletic development of a teratoma component [1]. These tumors may become malignant [9] as well as have a propensity for distant metastasis of borderline-appearing lesions [10]. Thus complete surgical excision is the treatment of choice. When the cysts are very large as in the case reported here, surgical excision may be fraught with important life-threatening complications. Rapid decompression of the venous compartment following brisk drainage of the cyst may result in fatal functional hypovolemia and a low cardiac output state [11].

## Conclusion

Large intra-abdominal mucinous cystadenomas may pose a diagnostic conundrum regarding their origin. Histopathological examination is inadequate in terms of differentiation between ovarian and extra-ovarian mucinous cystadenomas.
